# Phthalocyanines prepared from 4,5-dihexylthiophthalonitrile, a popular building block

**DOI:** 10.55730/1300-0527.3582

**Published:** 2023-10-03

**Authors:** Derya TOPKAYA, Zeynel ŞAHİN, Ümit İŞCİ, Fabienne DUMOULİN

**Affiliations:** 1 Department of Chemistry, Faculty of Sciences, Dokuz Eylül University, İzmir, Turkiye; 2 Department of Metallurgical & Materials Engineering, Faculty of Technology, Marmara University, İstanbul, Turkiye; 3 Department of Biomedical Engineering, Faculty of Engineering and Natural Sciences, Acıbadem Mehmet Ali Aydınlar University, İstanbul, Turkiye

**Keywords:** Phthalocyanine, phthalonitrile, hexylthio

## Abstract

Phthalocyanines are tetrapyrrolic artificial porphyrinoids that play major roles in advanced biological and technological applications. Research on this family of dyes is particularly active in Türkiye, with many derivatives being prepared from 4,5-dihexylthiophthalonitrile **DiSHexPN**, which is one of the most popular noncommercially available building blocks for phthalocyanines. This review summarizes the phthalocyanines and their versatile properties and applications that have been published since 1994, when the synthesis of **DiSHexPN** was first described, to emphasize the importance of this building block in plentiful applications, all with biomedical or technological impact.

## 1. Introduction

**Figure 1:**
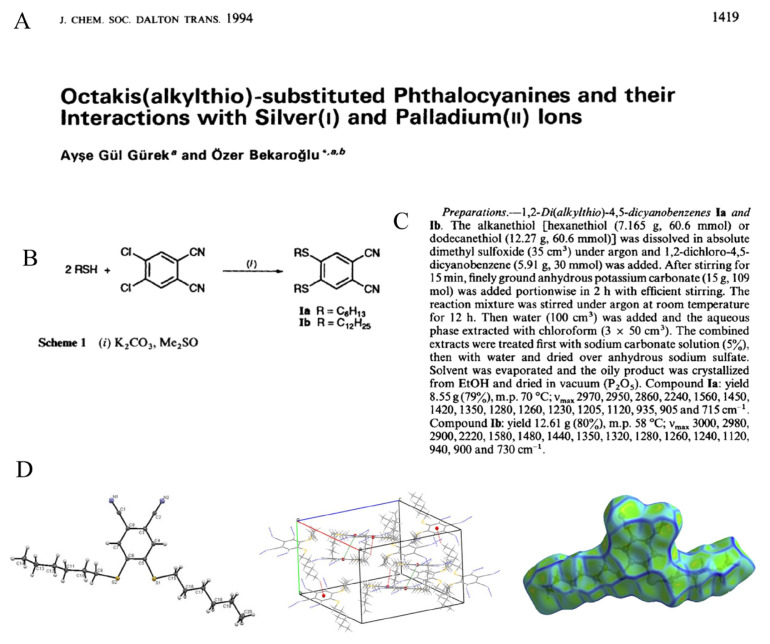
A: Reproduction of the title part of the original article describing the synthesis of **DiSHexPN**. B: Reproduction of scheme showing the synthesis of **DiSHexPN** in the same first article. C: Reproduction of the experimental part describing the synthesis and purification of **DiSHexPN** in the same first article. D: Left: Molecular structures of **DiSHexPN**. Middle: View of the unit cell with the dotted lines showing the intermolecular interactions. Right: Perspective view of Hirshfeld surface curvedness.

Many phthalocyanines are synthesized from commercially available phthalonitriles, the currently most popular building block for this family of artificial tetrapyrrolic porphyrinoid compounds. Pristine phthalonitrile and 4-tert-butyl-phthalonitrile may be the most commonly used, and are directly employed in the cyclotetramerization reaction leading to the formation of the phthalocyanine macrocycle. 4-Nitrophthalonitrile, 3-nitrophthalonitrile, and 4,5-dichlorophthalonitrile are also extremely widely used in phthalocyanine synthesis, but as intermediates to prepare substituted phthalonitriles. This review will focus on the role of 4,5-dihexylthiophthalonitrile for multiple reasons:- the availability of hexanediol and its relative affordability;

- the excellent solubility of resulting symmetrically substituted phthalocyanines in a large range of organic solvents, including hexane, and its low polarity allowing straightforward chromatographic purifications (unlike analogous alkoxy substituted phthalocyanines [1]);
- the commercial availability of many other alkanethiols, allowing the preparation of series of phthalocyanines to explore the effect of chain length on various properties;
- it may be the noncommercially available most widely used phthalonitrile. This assertion may be difficult to prove, but we identified 70+ publications that deal with this phthalonitrile or with phthalocyanines prepared using it. No other phthalonitrile amongst the different structures tested in SciFinder has been shown to be related to so many references;
- unlike phthalocyanine prepared using a monosubstituted phthalonitrile, the huge majority of the phthalocyanines prepared from 4,5-dihexylthiophthalonitrile exist as single compounds instead of regioisomeric mixtures. This is also true for phthalocyanines prepared from 4,5- and 3,6-disubstituted phthalonitriles.
- the fact that amongst the 70+ publications dealing with our title compounds, 60+ are from Turkish authors, including the first ever reported (Figure 1), reflecting that 4,5-dihexylthiophthalonitrile is a popular phthalocyanine building block, especially in Türkiye, which makes the topic especially relevant for this special issue.

## 2. Synthesis of 4,5-dihexylthiophthalonitrile

The reaction of hexanethiol with 4,5-dichlorophthalonitrile was first reported by Profs. Ayşe Gül Gürek and Özer Bekaroğlu in a seminal paper published in J. Chem. Soc. Dalton Trans. in 1994 (Figure 1A). Since then, this paper appears to have been cited a remarkable 184 times in the Web of Science as shown by a search conducted on 16 April 2023 [2]. 4,5-Dihexylthiophthalonitrile (**DiSHexPN**) was prepared in 79% yield in DMSO using potassium carbonate as a base, as shown by the reproduction of the synthesis pathway in Figure 1B and the reproduction of the description of the experimental conditions from the original article in Figure 1C. This work followed the report of the synthesis of 4,5-dichlorophthalonitrile [3] and its reaction with phenols, thiophenol and 1-propanethiol, the latter giving 4,5-propylthiophthalonitrile in 51% yield only. Since this initial report, the crystallographic structure of 4,5-dihexylthiophthalonitrile, including its Hirshfeld surface (Figure 1D), as well as a complete description of its spectroscopic properties in comparison with other alkanethio substituents has been published [4,5].

Even though phthalonitriles are usually reported as precursors of phthalocyanines, they exhibit intrinsic properties and **DiSHexPN** was found to exhibit good inhibition of stainless-steel corrosion, yet with a performance slightly lower than that of a related phthalonitrile with a diethylamino-ethanethio substituent [6].

## 3. Symmetrically substituted MPc(SHex)8

**Figure 2:**
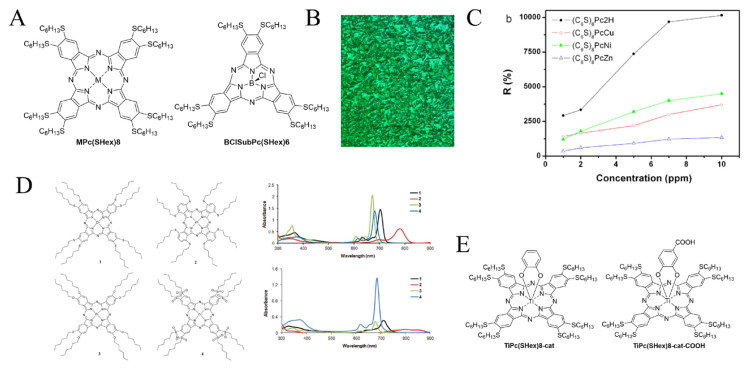
A: General structure of **MPc(SHex)8** phthalocyanines. B: Microphotograph of the texture adopted by **CuPc(SHex)8** observed under crossed polarizers. C: The dependence of sensor response on concentrations of NO_2_ for all **MPc(SHex)8** [M = 2H, Ni, Cu, Zn] thin films at 150 °C. D: Left: structure of **MPc(SHex)8** and three other phthalocyanines, Right: their UV-Vis absorption spectra in DCM and THF. E: Structures of **TiPc(SHex)8** phthalocyanines.

**DiSHexPN** was used firstly to synthesize symmetrically substituted **MPc(SHex)8** with M: 2H, Zn, Ni, Cu, Co, Mn, and Ti (Figure 2A). It was also used to prepare the symmetrically substituted subphthalocyanine **BClSubPc(SHex)6**.

The first publication [2] immediately reported nearly all the existing **MPc(SHex)8**, with M: 2H, Zn, Ni, Cu, Co, in the now classical DBU/pentanol conditions, and using MCl_2_ or M(OAc)_2_ metal salts. Yields were excellent, around 40% for all phthalocyanines, reflecting the high reactivity of **DiSHexPN**. Spectroscopic investigations in various organic solvents demonstrated the existence of axial interactions between the sulfur atoms of one phthalocyanine with the central metal of another one, for **CuPc(SHex)8** and **NiPc(SHex)8**, which is supposed to be enhanced by the vicinal position of the two thioethers substituents compared to tetrasubstituted SR phthalocyanines. To further explore the spectroscopic behavior of these first **MPc(SHex)8**, their interactions with Ag^+^ and Pd^2+^ ions were monitored. The presence of Ag^+^ reinforced the aggregation, whereas the introduction of Pd^2+^ disrupted it entirely at a Pd^2+^/phthalocyanine ratio. The complete electrochemical characterization of the **2HPc(SHex)8**, **CuPc(SHex)8**, and **ZnPc(SHex)8** was performed shortly after this first report [7].

Next, another crucial feature of **MPc(SHex)8** was identified, which is their ability to form thermotropic liquid crystalline discotic columnar mesophases, first by the **2HPc(SHex)8**, **CuPc(SHex)8**, and **CoPc(SHex)8** derivatives [8]. Their transition temperatures were lower than those of their alkoxy counterparts and inversely proportional to the chain lengths. The clearing point could not be determined as the liquid crystalline materials remained stable up to 300 °C but then decomposes instead of melting. Thin films could then be obtained by annealing of the **NiPc(SHex)8** (as well as the C12 analogues), and Raman spectroscopy was used to establish the dependence of film orientation on the temperature of spin-coated phthalocyanine films. It clearly showed that well-ordered films are obtained upon heating when the phthalocyanines have a disordered arrangement after the initial spin-coating/solvent evaporation [9]. Similarly, the molecular rearrangement of **CuPc(SHex)8** that has been spin-coated into thin films led to very organized films with clear mesogenic patterns (Figure 2B) [10].

Such organized thin films are advantageous for many applications [11]. One of them is the monitoring of protein absorption for biosensors. The adsorption of lysozyme, bovine serum albumin (BSA), and immunogamma globulin (IgG) onto organized films of **CuPc(SHex)8** has been monitored by quartz crystal microbalance dissipating monitoring (QCM-D), showing that the adsorption rate was faster for lysozyme than for BSA and IgG, but led to more rigid materials, whereas the adsorption of BSA and IgG is achieved with larger energy dissipation and results in water-rich soft multilayers, due to the protein hydration state [12,13]. Comparative experiments of the adsorption of BSA onto films of **CuPc(SHex)8** or **NiPc(SHex)8** showed that it is more limited onto **NiPc(SHex)8**, which was correlated to their surface wettability determined by contact angle measurements [14].

Phthalocyanines, as other porphyrinoids [15], are also known to be good chemical sensors [16–18], including against nitric oxide, which is a major air pollutant as well as a biomarker of health issues [19]. Thin films of **2HPc(SHex)8**, **NiPc(SHex)8**, **CuPc(SHex)8**, and **ZnPc(SHex)8** were tested against vapors of NO_2_ in different amounts to evaluate their potential as chemoresistive sensors. They exhibited excellent sensor response and response time and low temperature operation, with **2HPc(SHex)8** showing the maximum sensor response (Figure 2C). Thin films of **CuPc(SHex)8** coated onto an interdigital transducer (IDT) were tested against nitric oxide and ozone with or without oxygen doping. It was shown that the oxidizing gases could be desorbed by annealing the thin films [20].

Another way to arrange phthalocyanines at the molecular level is to prepare self-assembled monolayers (SAM). The presence of sulfur atoms was used to promote interactions onto gold electrode surfaces. The resulting devices coated with electrochemically active **CoPc(SHex)8** and **MnPc(SHex)8** showed good potential for the detection of H_2_O_2_, which paved the way for the development of enzyme activity monitoring such as glucose oxidase, which produces H_2_O_2_ as a subproduct of the conversion of glucose into gluconolactone [21].

In addition to their ability to self-assemble, **MPc(SHex)8** phthalocyanines have a red-shifted maximum absorption at far-red wavelengths, compared to other substitution patterns (except the octa SHex nonperipheral) or the unsubstituted ones (Figure 2D) [22].

**ZnPc(SHex)8** has been used for many spectroscopic works. Embedded into self-assembled peptide nanofibers, it gave nanomaterials with ultrafast intermolecular energy transfer [23]. Due to its triplet state properties prompted by its efficient ISC, **ZnPc(SHex)8** in a condensed phase made of plasticized gelatin fibers was used for far-red triplet sensitized molecular solid-state photoswitching of azobenzene [24].

The already red-shifted maximum absorption of classical **MPc(SHex)8** can be further pushed to NIR wavelengths with appropriate metalation, such as Ti metalation, which is especially efficient to red-shift the maximum absorption of **MPc(SHex)8** phthalocyanines. **TiPc(SHex)8-cat** with an axial catechol ligand exhibited a remarkable maximum absorption at 739 nm [25], the carboxylic acid functionalized **TiPc(SHex)8-cat-COOH** having a Q band at 732 nm in DMSO (Figure 2E) [26]. The peculiar photoproperties of **TiPc(SHex)8** prompted research towards more efficient synthetic methods, such as solvent-free conditions [27]. **DiSHexPN** was used in a tetramerization reaction in the presence of urea and Ti(O*^i^*Pr)4 salt to yield **O=TiPc(SHex)8** in high yields ranging from 16% to 42%, without the formation of **2HPc(SHex)8**, which usually complicates purification procedures.

Finally, more specific studies of the magnetic behavior of **CuPc(SHex)8** [28] and of its photoconductivity when included in TiO_2_ nanotube-**CuPc(SHex)8** heterojunction [29] have been reported.

## 4. Monomeric asymmetric substitution patterns

**Figure 3:**
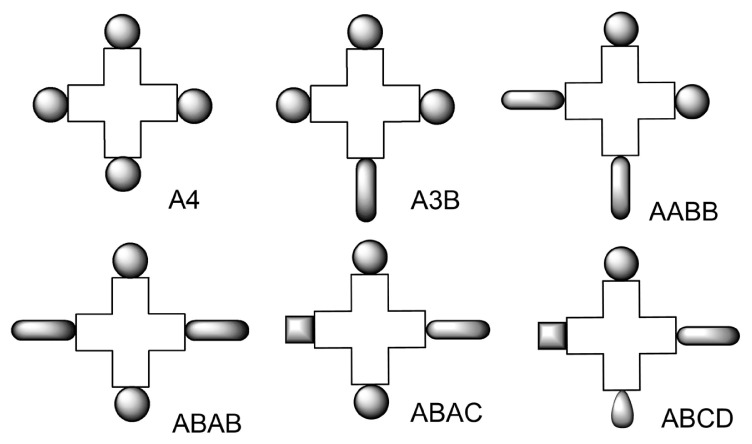
The different substitution patterns of phthalocyanines.

Phthalocyanines are composed of four different subunits. On each of these subunits, there are four positions likely to be substituted, with different grafting atoms or functions, and possibly functionalization or active moieties. Each subunit can have the same substituents or different ones; hence different substitution patterns have been defined (Figure 3). If A_3_B phthalocyanines are common, A_2_B_2_ phthalocyanines are rarer, and only a handful of ABAC [30] and ABCD [31] phthalocyanines have been reported.

Synthetic methods to access asymmetrically substituted phthalocyanines are based either on the statistical mixture of two precursors, which often implies tedious chromatographic separation steps, or on more selective methods that have other drawbacks, such as limited yield and/or access to the precursors [32].

A_3_B derivatives can be prepared by a statistical mixture of two phthalocyanine precursors, preferentially of the same reactivity, and which are usually phthalonitriles, or by preparing the subphthalocyanine of the A substituent and reacting it with the diiminoisoindoline of the B substituent [33]. This latter method leads to the free-base phthalocyanine (which can further undergo a metalation reaction) and is claimed to be selective but phthalocyanines with other substitution patterns have been observed after such a reaction.

Due to its advantages mentioned above, the diSHex substituent has been widely used to prepare various asymmetrically substituted phthalocyanines, which will be displayed in several subsections below: monomeric asymmetrically substituted with two different substituents, monomeric asymmetrically substituted phthalocyanines conjugated to another active moiety or material, dimeric phthalocyanines, trimeric phthalocyanines, and other multimeric constructs.

### 4.1. Monomeric asymmetrically substituted phthalocyanines with two different substituents

**Figure 4:**
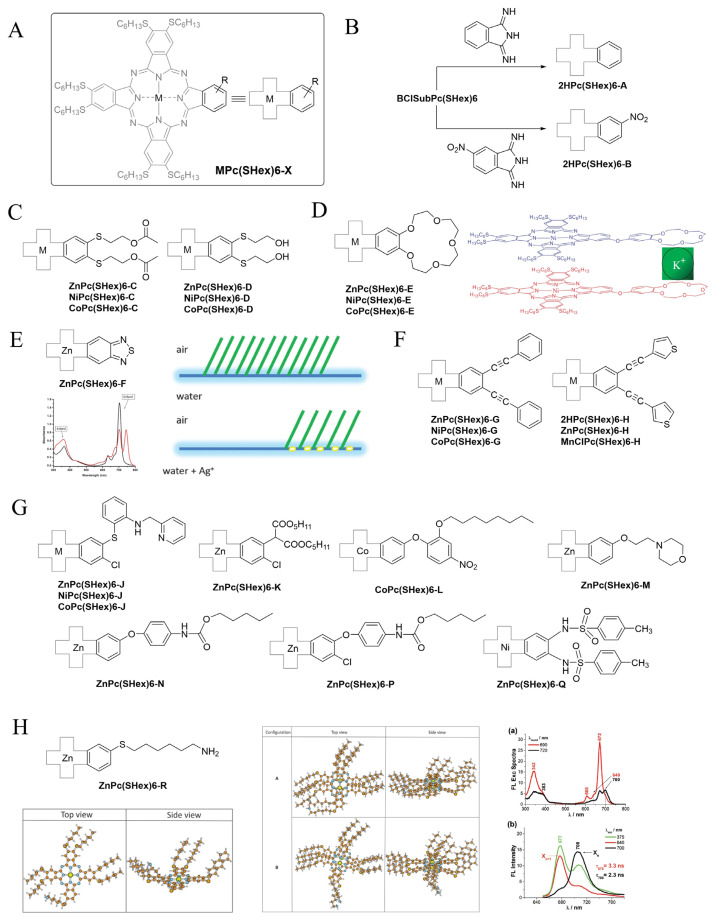
A–H: General structure of A_3_B asymmetrically **MPc(SHex)6-X** phthalocyanines. H Structure of phthalocyanine **ZnPc(SHex)6-R** in its electronic ground state, and lowest total energy configurations of the dimers. Excitation spectra (a) and fluorescence spectra (b) in DCM.

Except for one exception (to the best of our knowledge), all the monomeric asymmetrically substituted phthalocyanines with two different substituents—one of them being diSHex—are of the A_3_B type, and their schematic structure is represented in Figure 4A, with the general abbreviation being **MPc(SHex)6-X**. The first derivatives ever prepared were synthesized via the subphthalocyanine opening method, having **BClSubPc(SHex)6** reacting with diiminoisoindoline (aka isoindoline-1,3-diimine) or 5-nitroisoindoline-1,3-diimine (Figure 4B), giving **2HPc(SHex)6-A** and **2HPc(SHex)6-B**. The nitro function is a potential precursor of amine function, and a similar phthalocyanine with SC_12_H_25_ chains successfully underwent reduction for subsequent amidification reactions [34]. All the other monomeric A_3_B phthalocyanines have been prepared via the statistical method, by reacting a mixture of two precursors. Chromatographic purifications were readily possible thanks to the difference in polarity between **MPc(SHex)8** and **MPc(SHex)6-X**.

Two phthalocyanines resulting from a reaction between **DiSHexPN** and 1,2-(hydroxyethylthio)-phthalonitrile (pristine or modified) have been reported (Figure 4C). Acetylated 1,2-(hydroxyethylthio)-phthalonitrile reacted with **DiSHexPN** to yield **MPc(SHex)6-C** (M: Zn, Ni, and Co) [35]. The reaction was performed in anhydrous DMF, avoiding potential transesterification by using pentanol or hexanol as the solvent. 1,2-(Hydroxyethylthio)-phthalonitrile reacted with **DiSHexPN** to yield **MPc(SHex)6-D** (M: Zn, Ni, and Co) in similar conditions [36].

**MPc(SHex)6-E** (M: Zn, Ni, and Co) were obtained from 1-[(benzo-15-crown-5)-4-yl]oxyphthalonitrile [37] and **DiSHexPN** [38]. Titration experiments with alkali metal ions (Na^+^ and K^+^) of **ZnPc(SHex)6-E** and **NiPc(SHex)6-E** were performed by potentiometry measurements and revealed that the ions induce dimerization of the phthalocyanines, especially efficient when K^+^ is used (Figure 4D).

**ZnPc(SHex)6-F** is the only example that was prepared with a statistical mixture of **DiSHexPN** and of a diiminoisoindoline bearing the 1,2,5-thiadiazole, yielding a push–pull derivative that complexes Ag^+^ ions as demonstrated by titration experiments. The phthalocyanine is also amphiphilic, and the presence of the Ag^+^ ions in the water subphase induces a tilt of the self-ordered phthalocyanines at the air–water interface, promoting the formation of J-aggregates (Figure 4E) [39].

Alkynyl functions inserted directly at the periphery of the phthalocyanine macrocycle enhance the electronic delocalization and induce of red-shift of the maximum absorption, an effect that is further increased when another conjugated moiety is present. **MPc(SHex)6-G** (M: Zn, Ni, and Co) [40] and **MPc(SHex)6-H** (M: 2H, Zn, and MnCl) [41] (Figure 4F) have been prepared using the corresponding dialkynyl-substituted phthalonitriles, which were themselves obtained via Sonogashira coupling performed on 4,5-dichlorophthalonitrile.

Other A_3_B phthalocyanines have been prepared with various B substituents and metals (Figure 4G). **MPc(SHex)6-J** (M: Zn, Ni, and Co) have a bulky 6-[2-(2-pyridylmethylamino)phenylthiol substituent [42]. **ZnPc(SHex)6-K** has been prepared via mixed cyclotetramerization of 4-chloro-5-(1,1-dicarbethoxymethyl)-phthalonitrile [43] and **DiSHexPN** [44]. This asymmetric phthalocyanine proved to be thermically more stable than the reference **ZnPc(SHex)6**. **CoPc(SHex)6-L** has a 4-nitro-2-(octyloxy)phenoxy substituent and showed thermally activated conductivity dependence [45].

**ZnPc(SHex)6-M** is substituted by a morpholine moiety [46] that is known to potentiate the photodynamic activity of phthalocyanine [47–49]. It offers also the possibility of further quaternization, which is very useful for antibacterial photodynamic therapy. **ZnPc(SHex)6-M** exhibited promising properties for photodynamic therapy. **ZnPc(SHex)6-N** and **ZnPc(SHex)6-P** were prepared from 4-tert-butylcarbamatephenoxy-substituted phthalonitrile, with the substituent undergoing transcarbamoylation during the mixed cyclotetramerization reaction that was performed in pentanol [50]. **ZnPc(SHex)6-Q** carried a N,N′-ditosyl-o-phenylenediamine substituent and did not exhibit liquid crystalline thermotropic properties, unlike related derivatives with longer alkanethiol chains [51].

Aminophthalocyanine **ZnPc(SHex)6-R** was prepared by a series of successive reactions performed on a monohydroxylated phthalocyanine that was mesylated, underwent a nucleophilic reaction by sodium azide, and finally a Staudinger reaction yielding the primary amine function. Photoproperties were studied in THF, dichloromethane, and DMSO, in which the aggregation of **ZnPc(SHex)6-R** differs. The formation of highly emissive H-aggregates in all solvents could be demonstrated spectroscopically and confirmed by theoretical calculations. Lifetimes up to 48 ns were observed (Figure 4H) [52].

**Figure 5:**
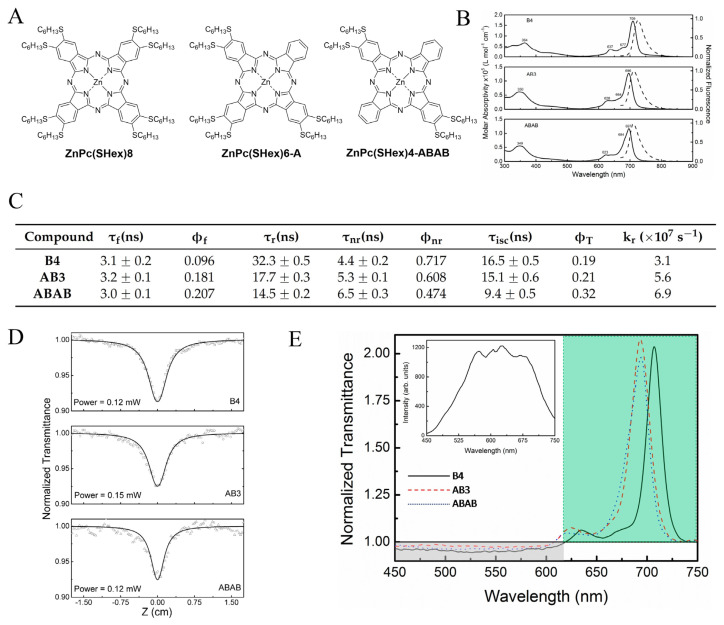
A: Structure of the 3 phthalocyanines of the series (**ZnPc(SHex)4**, **ZnPc(SHex)6-A** and **ZnPc(SHex)4-ABAB**). B: Molar absorption (solid lines) and normalized fluorescence (dashed lines) spectra of **ZnPc(SHex)4**, **ZnPc(SHex)6-A** and **ZnPc(SHex)4-ABAB** in chloroform. C: Photophysical parameters of the 3 phthalocyanines in chloroform. D: Normalized transmittance obtained by the single pulse Z-scan at 532 nm (symbols), and the theoretical fitting (solid line). E: Normalized transmittance spectra obtained by using the WLCZS; inset: the white light continuum spectrum used in the WLCZS technique.

Finally, the nonlinear and other spectroscopic properties of a series of 3 phthalocyanines (Figure 5A) composed of **ZnPc(SHex)6**, of the A_3_B derivative **ZnPc(SHex)6-A**, and of the crosswise asymmetric **ZnPc(SHex)4-ABAB** have been comparatively studied in chloroform [53]. The cross-section of each phthalocyanine’s singlet and triplet excited absorption excited states has been determined by single pulse and train-pulse Z-scan techniques at 532 nm, as well as the fluorescence lifetime by time-resolved fluorescence experiments. The Q band maximum of **ZnPc(SHex)6-A** and **ZnPc(SHex)4-ABAB** in chloroform is similar, blue-shifted by 10 nm compared to **ZnPc(SHex)8** (Figure 5B). The influence of the asymmetry of the substitution pattern is much more linear on the fluorescence quantum yields than on other spectroscopic parameters (Figure 5C). This more proportional trend is also observed for the singlet excited state absorption cross-section values, 2.8 times higher than the grounds state absorption for **ZnPc(SHex)6-A**, 3.2 times higher for **ZnPc(SHex)6-A**, and 4 times higher for **ZnPc(SHex)4-ABAB** (Figure 5D). Experiments with the white light continuum Z-scan (WLCZS) technique showed the same trend, as seen in Figure 5E.

### 4.2. Monomeric asymmetrically substituted phthalocyanines conjugated to another active moiety or material

While the outstanding properties of phthalocyanines, especially their photoproperties, can be exploited in technological applications [54,55], this often requires them to be combined with other active moieties or materials. Having DiSHex substituents on three of the four isoindole subunits confers good solubility and facilitates the introduction of a functionalization and facilitates the introduction of a functionalization on the fourth subunit, together with relatively easy chromatographic purifications.

Photodynamic therapy is the most common biomedical application for phthalocyanines. The efficiency can be improved by using amphiphilic photosensitizers or targeting units, which is why many phthalocyanines have been conjugated to biomolecules [56].

**Figure 6:**
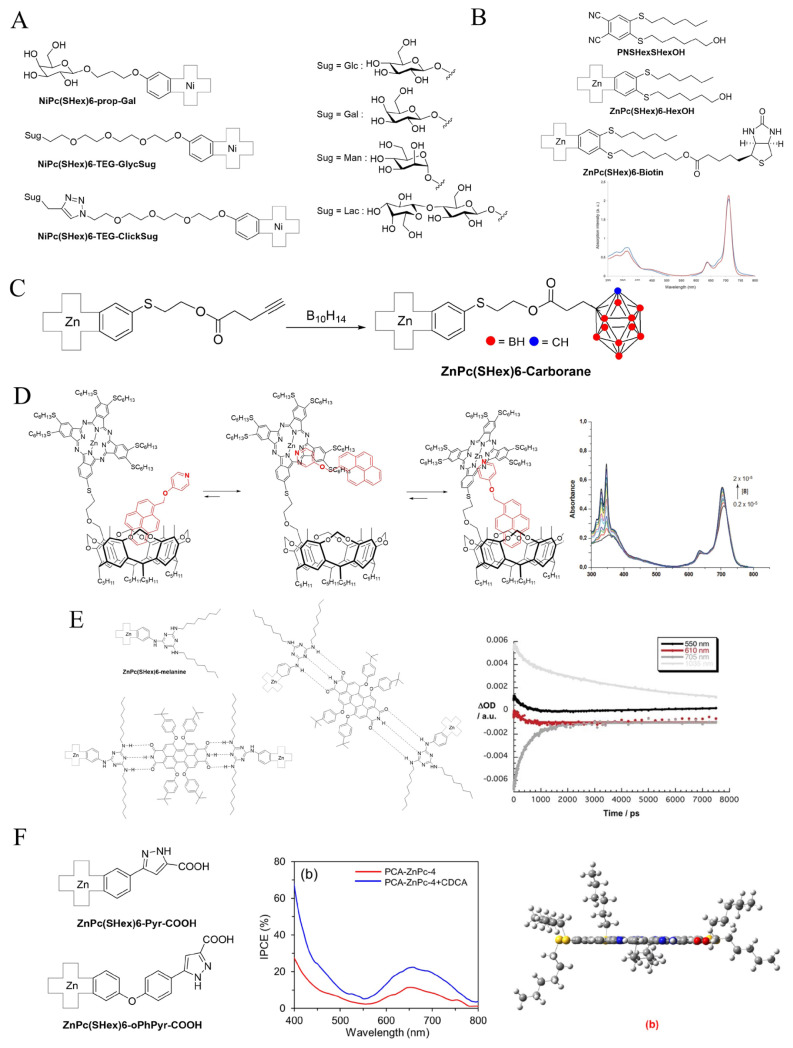
A: Structure of the directly glycosylated and clicked carbohydrate-functionalized phthalocyanines. B: Structure of **PNSHexSHexOH**, **ZnPc(SHex)6-HexOH**, and **ZnPc(SHex)6-Biotin**. Superposition of the UV-Vis electronic absorption spectra of **ZnPc(SHex)8** (blue) and **ZnPc(SHex)6-HexOH** (red) at 10 μM in chloroform, evidencing the same maximum of their Q band. C: Synthesis of **ZnPc(SHex)6-Carborane**. D: Left: Structure of the **ZnPc(SHex)6-Resorc** and the different interactions possibilities with pyrene–pyridine. Right: UV-Vis titration spectra of **ZnPc(SHex)6-Resorc** with the pyrene–pyridine guest. E: Left: Structure of the SYN and ANTI 2+1 **2HPc(SHex)6-melanine**•**PDI** constructs. Right: time absorption profiles at 550, 610, 705, and 1035 nm, monitoring the charge separation and charge recombination dynamics. F: Left: Structure of the two pyrazole–COOH phthalocyanines **ZnPc(SHex)6-Pyr-COOH** and **ZnPc(SHex)6-OPhPyr-COOH** used in DSSC devices. Middle: IPCE spectra of the **ZnPc(SHex)6-Pyr-**COOH-DSSCs with and without CDCA. Right: Theoretically optimized side view of **ZnPc(SHex)6-Pyr**.

The conjugation of phthalocyanines to carbohydrates via either classical glycosylation or click chemistry has been reported onto Ni phthalocyanines (Figure 6A) [57,58]. The preparation of this series of conjugates is an example of how functionalized phthalocyanines can be engaged in several further reactions instead of being the ultimate step of a synthetic sequence. Monohydroxylated phthalocyanines with a propyloxy or a tetraethylene glycoloxy spacer were prepared, the latter also being converted into the azido analogue. Direct glycosylation of the monohydroxy phthalocyanines behaving as the glycosylation acceptor could be performed in remarkable yields using benzoylated glycopyranosyl trichloroacetimidate donor (with β-glucose, β-galactose, α-mannose, and β-lactose carbohydrate skeleton). **NiPc(SHex)6-prop-Gal** and **NiPc(SHex)6-TEG-GlycSug** were obtained in up to 90% yields following procedures optimized previously for others glycoconjugates, followed by an ultimate carbohydrate deprotection reaction [59–61]. It is worth noting that these reactions are performed at 0 °C, and that the excellent solubility conferred by the six SHex chains was crucial to be able to engage the phthalocyanines in such glycosylation reactions. As previously mentioned for the synthesis of **ZnPc(SHex)6-R**, intermediate monoazido phthalocyanine could be readily prepared and used in a click reaction with the same carbohydrates in their acetylated propargylated form, the click reaction being performed in biphasic conditions in vigorously stirred dichloromethane/water mixtures, using sodium ascorbate and copper sulfate pentahydrate as the CuAAC promoters [62]. **NiPc(SHex)6-TEG-ClickSug** were finally obtained after a last deprotection reaction.

Because photoproperties are very sensitive to substitution pattern variation [63], it was noted that these heptasubstituted derivatives have a slightly blue-shifted maximum absorption compared to the **MPc(SHex)8** that are usually used as the references for spectroscopic measurements. **PNSHexSHexOH** has been prepared by reacting 4,5-dichlorophthalonitrile with two different thiols. Mixed cyclotetramerization of **PNSHexSHexOH** with **DiSHexPN** gave **ZnPc(SHex)6-HexOH**, which had exactly the same maximum absorption as **ZnPc(SHex)8**. Further functionalization by esterification with biotin gave **ZnPc(SHex)6-Biotin**, a vitamin useful for cancer cell targeting (Figure 6B).

Also for biomedical applications, an elegant theranostic combination of photodynamic therapy and boron neutron capture therapy (BNCT) dual agent has been envisioned when preparing the **ZnPc(SHex)6-Carborane** conjugate (Figure 6C) [64]. A monohydroxylated phthalocyanine could be successfully used in a reaction sequence, first Steglich esterification with pentynoic acid. Next, reaction with decaborane afforded the final **ZnPc(SHex)6-Carborane** conjugate in 40% yield.

In addition to photodynamic biomedical purposes, the photoproperties of phthalocyaniens are widely used for electron/energy transfer applications when they are combined with another spectroscopically photoactive molecule.

Maintaining pyrene and related aromatic molecules close to phthalocyanine to promote such electronic events has been performed with various covalent assemblies [65,66]. Another strategy is the use of supramolecular constructs. The phthalocyanine–resorcinarene hybrid (Figure 6D) [67] was designed to benefit from the pyrene-hosting capacity of the resorcinarene cavitand, while the pyridine axially interacts weakly with the Zn phthalocyanine macrocycle. Titration experiments using UV-Vis spectroscopy confirmed the role of the cavitand in the maximization of the phthalocyanine–pyrene interactions.

Further, with the purpose to maximize the interactions between a Zn phthalocyanine core and another photoactive moiety, this time perylenediimide, **2HPc(SHex)6-melanine** was prepared to establish hydrogen bonds with perylenediimine. Two **2HPc(SHex)6-melanine** established each triple hydrogen bond with perylenediimine, forming 2+1 **2HPc(SHex)6-melanine**•**PDI** constructs that can arrange in a SYN or an ANTI configuration (Figure 6E) [68]. Intramolecular electron transfer was observed as well as a PDI^−^/**2HPc(SHex)6-melanine**^+^ species with a lifetime of several nanoseconds.

Light harvesting for electric current generation in DSSC is another frequent application of phthalocyanines combined with materials such as titanium oxide [69]. Carboxylic acid functions are then necessary to anchor the phthalocyanines on TiO_2_. To this end, many phthalocyanines have been prepared since the famous TT1 [70]. Introduced on a **ZnPc(SHex)6** core, several derivatives using a pyrazole with a carboxylic acid function have been designed (Figure 6F) [71,72]. Incident photon-to-current conversion efficiency (IPCE) showed the superior properties of **ZnPc(SHex)6-Pyr-COOH** compared to **ZnPc(SHex)6-OPhPyr-COOH**, including when doped when chenodeoxycholic acid. The theoretically optimized structure of **ZnPc(SHex)6-Pyr-COOH** showed a predominantly planar geometry.

### 4.3. Dimeric

**Figure 7:**
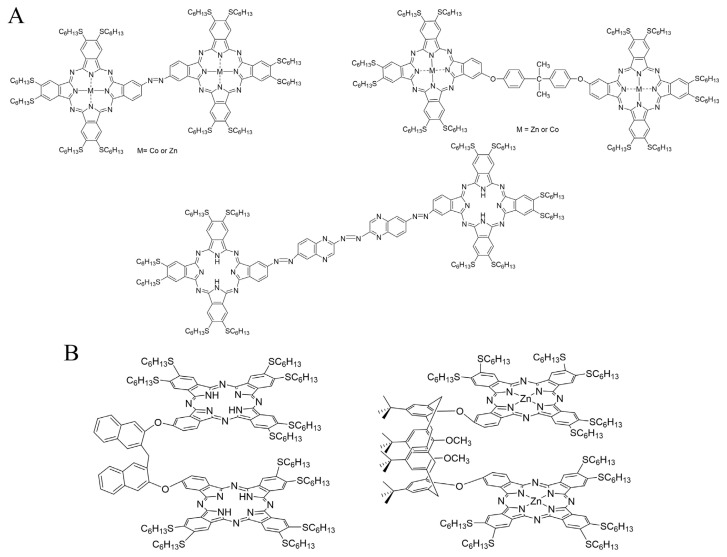
A: Structure of dimeric phthalocyanines. B: Structure of clamshell dimeric phthalocyanines.

Several dimeric complexes of phthalocyanines (Figure 7) with DiSHex substituents have been identified, and can be divided into two categories, depending on their general geometry imposed by their spacer. Either the two macrocycles are on the same axis, or the whole construct has a so-called clamshell shape when the spacer maintains the two phthalocyanines macrocycles on top of each other.

Three phthalocyanines with an axis-like spacer have been reported (Figure 7A). The azo-bridged one was prepared by the reaction of two mono-nitro phthalocyanines with powdered Zn in a NaOH/MeOH/THF solution that formed the azo bridge after reduction of the nitro function [73]. The same mono-nitro phthalocyanine precursor was used to react with 4-nitro-o-phenylenediamine to form a new A_3_B derivative, whose phenylenediamine function was converted into a dioxime that finally dimerized [74]. A more simple 4,40-isopropylidendioxydiphenyl bridged dimeric complex was formed by reacting a dimeric phthalonitrile with a large excess of **DiSHexPN** [75].

The two identified clamshell dimeric phthalocyanines were prepared from clamshell dimeric phthalonitriles, one with a 1,1′-methylenedinaphthalen-2-oxy spacer [76] and the other with a crosswise functionalized calixarene (Figure 7B) [77]. The resulting dimeric phthalocyanines exhibited UV-Vis spectra reflecting the internal aggregation of the two macrocycles closely maintained on top of each other.

### 4.4. Trimeric constructs

Different bridges have been used to form trimeric phthalocyanine constructs. It is interesting to note that the synthetic strategy was different depending on the type of bridge selected, either by preparing a functionalized A_3_B phthalocyanine reacting with the bridge precursor0 or grafting first three phthalonitrile on the bridging unit before reaction with **DiSHexPN**.

**Figure 8:**
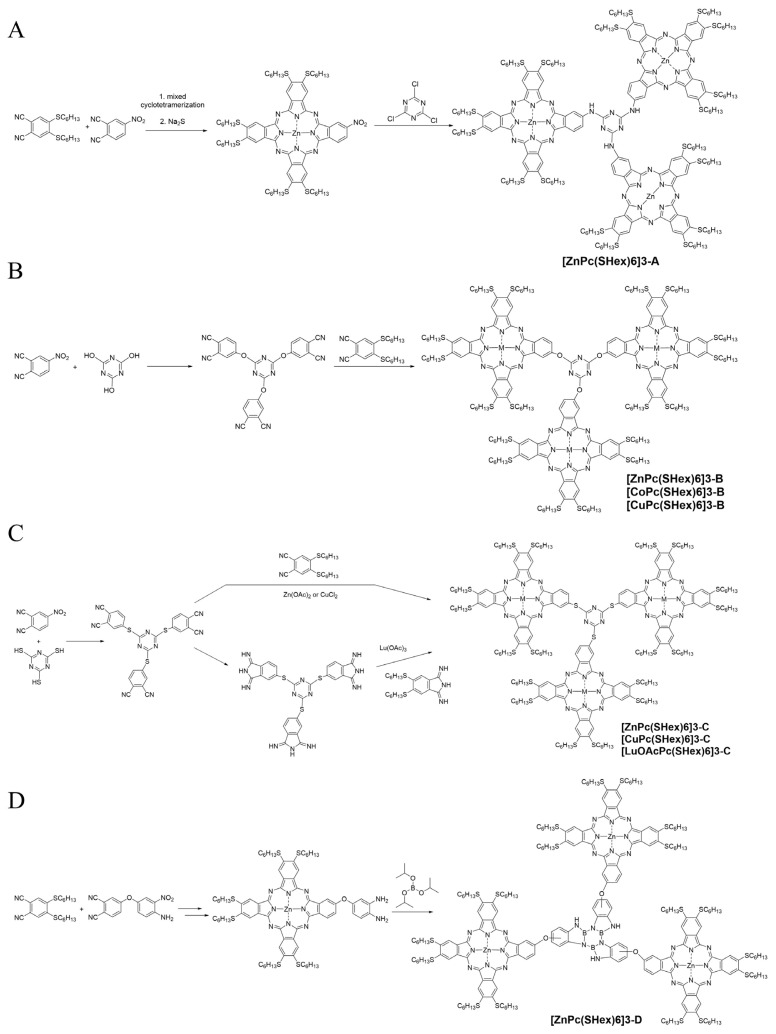
A: Synthesis of s-triazine-bridged trimeric phthalocyanine **[ZnPc(SHex)6]3-A** with NH grafting functions. B: Synthesis of s-triazine-bridged trimeric phthalocyanines **[MPc(SHex)6]3-B** with O grafting atom. C: Synthesis of s-triazine-bridged trimeric phthalocyanines **[MPc(SHex)6]3-C** with S grafting atom. D: Synthesis of borazine-bridged trimeric phthalocyanine **[ZnPc(SHex)6]3-D**.

The reaction of an A_3_B Zn phthalocyanine with three isoindole subunits having the diSHex substituent and the fourth subunit having a primary amine function with cyanuric chloride (Figure 8A) readily gave the trimeric construct **[ZnPc(SHex)6]3-A** with a triazine bridge [78]. Thanks to the SHex chains, aggregation in organic solvents was limited.

Three closely related trimeric constructs with an s-triazine bridging three oxygen-linked phthalocyanines [79] (Figure 8B) have been prepared by inserting first the phthalonitriles on the s-triazine, with the resulting trisphthalonitrile reacting with 10 equivalents of **DiSHexPN** in the presence of the metal salt (Zn acetate, Co chloride, or Cu chloride) to yield **[MPc(SHex)6]3-B** (M: Zn, Co, or Cu) that were slightly more aggregated.

Three other closely trimeric constructs, **[MPc(SHex)6]3-C** (M: Zn, Cu, or LuOAc), have been prepared, in which the phthalocyanines are linked to the s-triazine bridge with sulfur atoms. The Zn and Cu derivatives could be prepared by mixed cyclotetramerization of the trimeric phthalonitrile with **DiSHexPN**, but it was necessary to prepare the corresponding diiminoisoindoline derivatives to obtain the Lu(OAc) trimeric phthalocyanine (Figure 8C) [80].

Finally, the borazine-bridged trimeric construct **[ZnPc(SHex)6]3-D** (Figure 8D) [81] was prepared from an A_3_B diamino phthalocyanine reacting with triisopropoxyborane, and exhibited gas sensing properties quantified by electrochemical and electrical techniques. It was tested against a large range of organic solvents and proved to have excellent properties, in terms of both response time and high sensitivity, especially for chloroform, which is a volatile organic compound (VOC) of interest.

### 4.5. Other multimeric constructs

A tetrameric and two hexameric phthalocyanine constructs have been prepared and all have been used for gas sensing applications.

**Figure 9:**
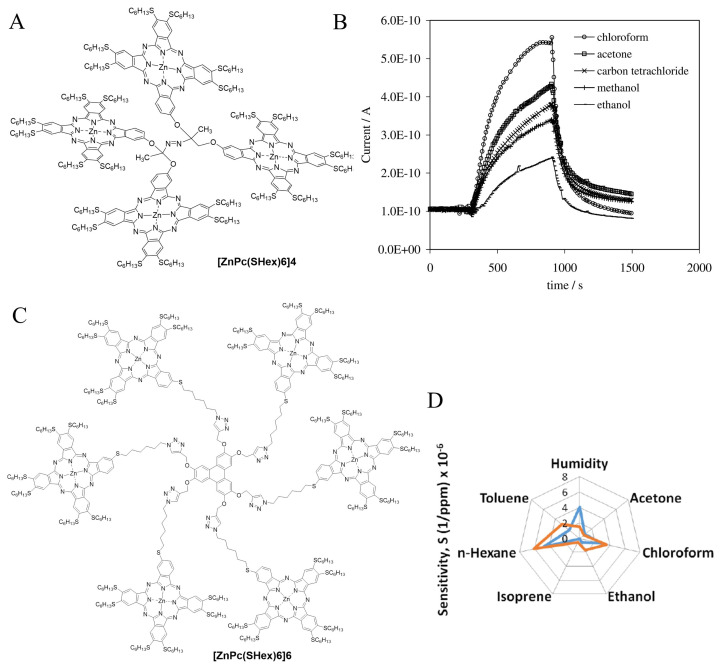
A: Structure of the tetrameric phthalocyanine construct **[ZnPc(SHex)6]4**. B: Current vs. time of a spin-coated **[ZnPc(SHex)6]4** film exposed to different VOC vapors (200 ppm). C: Structure of the hexameric phthalocyanine **[ZnPc(SHex)6]6p** with the spacer in peripheral position. D: Sensitivity of **[ZnPc(SHex)6]6p** with a nonperipheral (blue) and peripheral (orange) grafting point (radar representation).

**[ZnPc(SHex)6]4** and **[CoPc(SHex)6]4** (Figure 9A) were obtained when a dimeric construct bridged by 2-nitro-2-methyl-1,3-propanedioxy spacer reacted with powdered Zn in a Me/THF NaOH solution [82]. The sensing properties of the resulting **[ZnPc(SHex)6]4** and **[CoPc(SHex)6]4** were tested for various VOC vapors (Figure 9B). The d.c. conductivity experiments showed that the **[ZnPc(SHex)6]4** tetrameric construct had the best sensitivity for the VOCs as well as complete reversibility even at room temperature, which is a crucial parameter for such applications.

**[ZnPc(SHex)6]6p** and **[ZnPc(SHex)6]6np** were prepared by click chemistry, a powerful synthetic tool [83–85]. First, two mono-azido phthalocyanines, one being a precursor of **ZnPc(SHex)6-R** and the other one differing only by the nonperipheral position of the spacer, were prepared. They reacted with 2,3,6,7,10,11-hexakis(prop-2-ynyloxy)triphenylene in excellent yields (50%) given that actually six click reactions are performed concomitantly (Figure 9C). Both constructs were coated on surface acoustic wave (SAW) transducers via the electrospraying method and tested against acetone, ethanol, n-hexane, toluene, chloroform, and isoprene, and exhibited the best sensitivity and LOD values against toluene and ethanol vapors (Figure 9D).

## 5. Lanthanide double-decker and multidecker complexes

Phthalocyanines can form double-decker complexes with rare-earth metals, as well as triple and multiple complexes. Other tetrapyrrolic derivatives such as porphyrins can be part of these complexes. When all the macrocycles are the same, the complex is said to be homoleptic, and heteroleptic when the macrocycles are different, should it be only because of a different substitution pattern or because different macrocycles are present. With triple- and multideckers, different metals can be found on the whole structure. These complexes can be obtained directly during the cyclotetramerization reaction, or by engaging nonmetalated phthalocyanines in metalation reactions with the rare-earth metals salts. A simple double-decker can exhibit several electronic forms: reduced, neutral, and oxidized, which have specific magnetic, electrochemical, and spectroscopic properties. In particular, their single molecule magnet (SSM) behavior is very suitable for quantum computing and molecular spintronic technologies [86,87].

### 5.1. Lanthanide homoleptic double-decker complexes

**Figure 10:**
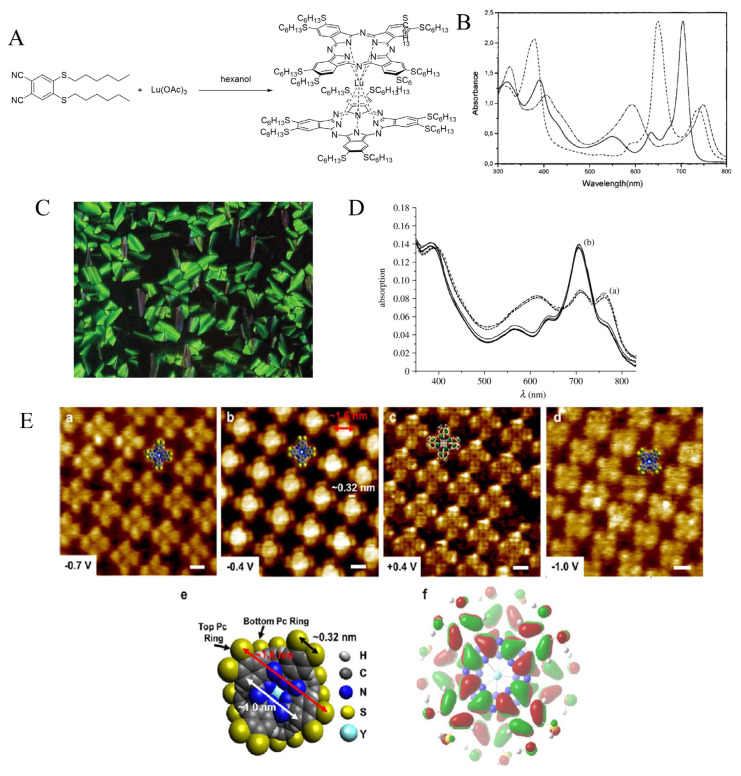
A: Structure of **[Pc(SHex)8]2Lu**. B. UV-Vis spectra of **[Pc(SHex)8]2Lu** in its differently oxidized forms: neutral in chloroform (continuous line), oxidized in chloroform (dash–dot line) and reduced in tetrahydrofuran (dash). C: Microphotograph of the mesogenic texture of **[Pc(SHex)8]2Sm**. D: UV-Vis spectra of spin-coated films of **[Pc(SHex)8]2Dy** after 1, 5, 15, and 20 cycles of oxidation by nitric acid vapors (a) and as-much interactions with NADH solutions (5 mM, phosphate buffer) (b). E: Submolecular resolution HR-STM images of **[Pc(SHex)8]2Y**-PO/HOPG (a–c) and **[Pc(SHex)8]2Y**-PO/Au(111) (d) interfaces. Structural model (e) and electron density distribution calculated by DFT (f) of **[Pc(SHex)8]2Y**.

The first homoleptic complex of octahexylthiophthalocyanine was reported by Profs. Ayşe Gül Gürek and Vefa Ahsen in a collaborative paper with a French team [88]. Lutetieum was used to complex two octahexylthio phthalocyanine macrocycles. The molecule was prepared directly from the phthalonitrile (2.68 g, 7.43 mmol, 6.5 equiv) with anhydrous Lu(OAc)_3_ (0.4 g, 1.14 mmol), 1,8-diazabicyclo[5.4.0]undec-7-ene (DBU) in hexan-1-ol, with a final yield of 11% of the crystalline desired complex (Figure 10A). The oxidized form can be obtained by adding bromine of the complex dissolved in chloroform, and the reduced form is generated by adding NaBH_4_ to a tetrahydrofuran solution of the molecule (Figure 10B). The NMR spectra of these two forms can be recorded when deuterated solvents are employed, while only the peaks corresponding to the alkyl signals can be obtained for the neutral form as it is magnetically active.

The X-ray single crystal structure of the neutral form could be obtained. It shows that the angle between the two macrocycle is 42°, near to that for unsubstituted complex (45°) [89], meaning that the presence of the hexylthio chains has no significant effect on the structure, which is chiral. The magnetic behavior was modeled and revealed an antiferromagnetic coupling along one-dimensional chain of spin S = 1/2 with *g* = 2.04. A full series was next formed with the synthesis of the GdIII, DyIII, and SmIII complexes, which were obtained in ~30% yield [90]. X-ray analysis revealed that the metal did not affect the angle between the two phthalocyanine rings.

Like the Lu complex, they crystallize in the monoclinic space group *C2/c*, and they all exhibit thermotropic mesogenic liquid crystalline behavior forming form columnar-hexagonal mesophases (Figure 10C).

Thin films could be easily obtained by film-coating technique. Thin films of the dysprosium complex were used to detect nicotinamide adenine dinucleotide hydride (NADH) in water solutions [91]. NADH reduces the complex into its reduced form, and the film sensing capacity is restored upon exposure to nitric acid (HNO_3_) vapors. The system remained stable even after 20 cycles (Figure 10D).

More recently, the yttrium complex was also prepared, in 24% yield, using this time another type of lanthanide salt (Y(acac)3·2H_2_O) with lower stoichiometry (8 equiv of salt relative to the phthalonitrile). The complex spontaneously adsorb onto 1- phenyloctane (1-PO)/highly oriented pyrolytic graphite (HOPG) or 1-PO/Au(111) interfaces by scanning tunneling microscopy (STM) with very high resolution (Figure 10E) [92]. Adsorption from micromolar solutions of phthalocyanine gave well-ordered layers at the 1-PO/HOPG interface, but structures with islands using less concentrated solutions. Due to the presence of sulfur atoms, the complex nucleated much more at the 1-PO/Au(111) interface, where it exhibited much mobility and hence ability to self-order.

### 5.2. Homoleptic complexes of ABAB

**Figure 11:**
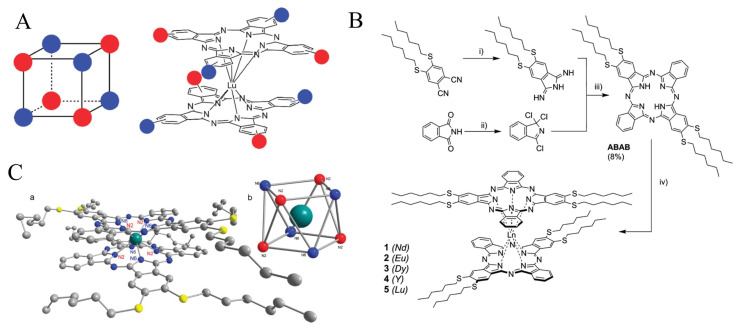
A: Schematic representation of a Ln double-decker showing its octupolar distorted cube shape. B: Synthesis of the **(ABAB)2Ln** complexes. C: X-ray crystallographic structure of **(ABAB)2Ln** and cubic-like coordination around the Lu atom.

The ideal molecular octupole is a cube with alternating charges at each corner and electronic interactions through the edges. Such objects have been conceptualized to have a noncentrosymmetry cancelling the molecule dipolar moment but yet a giant quadratic hyperpolarizability originating from their second-order nonlinear optical properties. Lanthanide complexes of phthalocyanines with an asymmetric ABAB substitution pattern appear suitable to realize such objects (Figure 11A), even if the cube would be slightly twisted due to the ~45° staggering angle between the two phthalocyanine rings [93,94]. The ABAB free-base phthalocyanines with crosswise diSHex substitution have been prepared from more unusual phthalocyanine precursors, using a selective synthetic method based on the use of a diiminoisoindoline carrying the two hexylthio chains, and 1,3,3-trichloroisoindolenine, a method previously used for similar derivatives [95–97]. The ABAB phthalocyanines were finally obtained using lanthanide acetate salt at high temperature (refluxing 1-chloronaphthalene) and DBU as a base (Figures 11B and 11C). Five different **(ABAB)2Ln** double-decker complexes were prepared: Lu, Eu, Nd, Y, and Dy.

Measurements of the dynamic molecular first hyperpolarizabilities by hyper-Rayleigh scattering (nonpolarized HLS) using a 1907 nm incident wavelength confirmed the relevance of the design and evidenced the role of the metal. The dynamic molecular first hyperpolarizability values ranged from 3010 × 10^−30^ esu for the yttrium complex to 5760 × 10^−30^ esu for the lutetium one, all being remarkably large for these measurement conditions. A correlation with the number of f electrons could also be established, as well as the reduced or oxidized state of the complexes.

### 5.3. Heteroleptic Ln complexes

Ln complexes in which at least one of the phthalocyanines has a DiSHex substituent are the topic of this subpart. Three synthetic pathways are possible for such complexes: i) reacting **DiSHexPN** with another phthalonitrile in carefully devised ratio in the presence of Ln salt, ii) reacting a formed monoLn phthalocyanine with phthalonitrile or diiminoisoindoline, or iii) reacting two free-base phthalocyanines with Ln salt.

**Figure 12:**
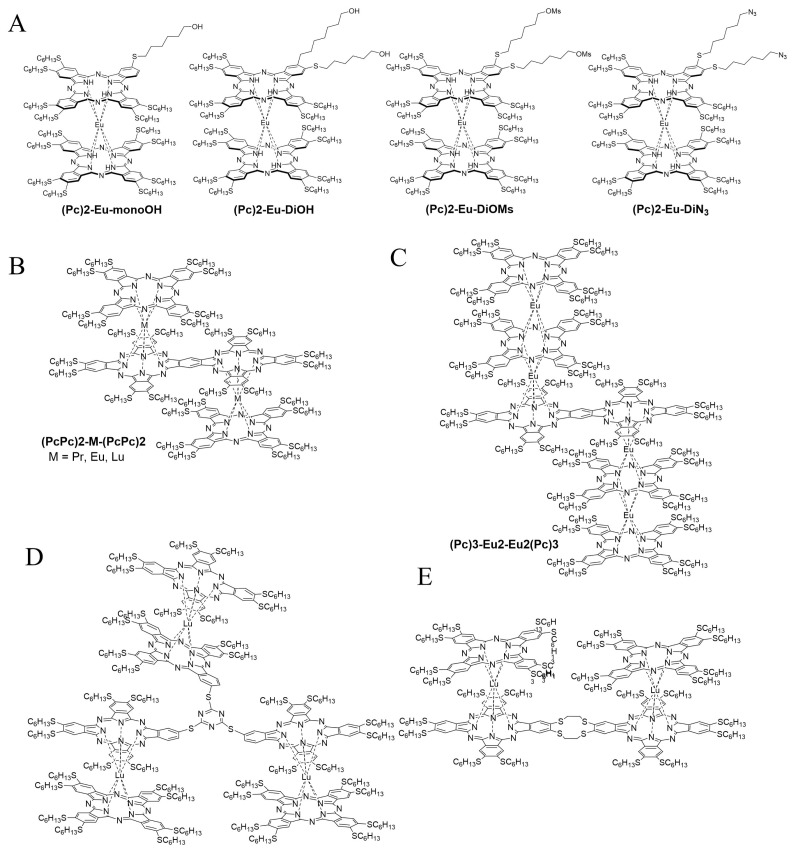
Structure of heteroleptic Ln double- and multideckers

The first strategy was applied to the preparation of **(Pc)2-Eu-monoOH** and **(Pc)2-Eu-diOH**, which are to the best of our knowledge the only functionalized double-deckers (Figure 12A) [98]. **(Pc)2-Eu-diOH** underwent further reactions to increase the range of available functionalization by being converted into the dimesylated and the di-azido complexes. **(PcPc)2-M-(PcPc)2** (Figure 12B) were also synthesized by the mixed cyclotetramerization of **DiSHexPN** and bis(diiminoisoindoline) in the presence of acac salts of lanthanides. The resulting complexes exhibited excellent nonlinear optical values [99]. **(Pc)3-Eu2-Eu2(Pc)3** was then obtained by reacting **(PcPc)2-M-(PcPc)2** with **DiSHexPN** and Eu(acac)3·nH_2_O in pentanol/DBU conditions to give this doubly triple-decker construct that exhibited remarkable performance in ambipolar organic field effect transistors (Figure 12C) [100]. The synthesis of the other tetrameric [101] Lu complex was performed by the second strategy, the free-based dimeric phthalocyanine being first activated by lithium metalation and then reacting with **DiSHexPN** (Figure 12D). The hexameric Lu complex (Figure 12E) was prepared following the third strategy, from the trimeric **[2HPc(SHex)6]3-C** trimeric construct reacting with **2HPc(SHex)6** [102].

One can guess that the purification of all the elaborated complexes requires thorough chromatographic steps and hence good solubility in organic solvents, which is conferred by the diSHex chains.

## 6. Disulfonylhexyl substituted phthalocyanines

Thioethers can be oxidized into sulfonyl functions, and this was done on alkanethiol-substituted phthalonitriles in 1996, with many works by the Torres group to prepare push–pull phthalocyanines for their nonlinear optical properties [103]. **DiSO_2_HexPN**, **MPc(SO_2_)8** and related asymmetrically substituted derivatives are prepared from **DiSHexPN** and hence are within the scope of this review.

**Figure 13:**
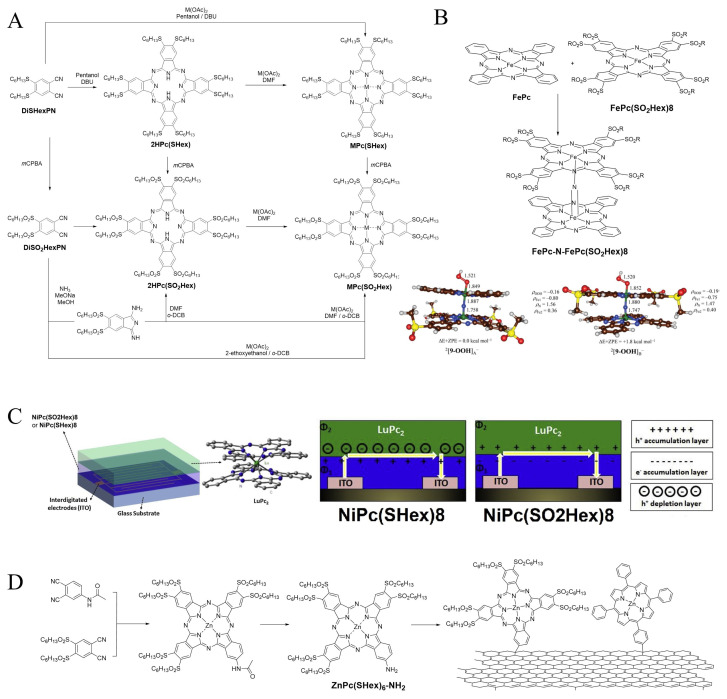
A: Synthetic pathways from **DiSHexPN** to **MPc(SO_2_)8**. B: Preparation of the heteroleptic *N*-bridged diiron phthalocyanine complex **FePc-N-FePc(SO2Hex)8**, and DFT optimized geometries of **FePc-N-FePc(SO2Hex)8**[9–OOH] at the B3LYP level of theory. Bond lengths are in angstroms and spin densities in atomic units. C: Bilayer heterojunction devices with **NiPc(SO2Hex)8** and **NiPc(SHex)8**, and the different charge transfer pathways at the interface of **NiPc(SO2Hex)8** and **NiPc(SHex)8**/LuPc2 heterojunctions. D: Preparation of **ZnPc(SHex)6-NH_2_** and grafting onto RGO with a porphyrin.

An overview of the possible synthetic pathways leading to **MPc(SO_2_)8** (free-base or metalated) was conducted by Jiang’s team in 2011 [104] and is presented in Figure 13A. The sulfonyl moiety has a very electrophilic sulfur atom that renders it prone to nucleophilic attacks when classical conditions such as Li/pentanol are used, which is the reason why basic conditions are not used. Yields are anyway satisfying due to the high reactivity of **DiSO_2_HexPN** in high-boiling point solvents. These phthalocyanines were used in microwire devices and the measured conductivities −5.24 × 10^−4^ S.m^−1^ for **2HPc(SO_2_)8**, 2.73 ×10^−4^ S.m^−1^ for **CuPc(SO_2_)8**, and 1.17 × 10^−7^ S.m^−1^ for **ZnPc(SO_2_)8** confirmed their potential.

The other advantage of using sulfonyl substituents is that the sulfur atom is at its maximum oxidation state, which is especially suitable for oxidation catalysts. *N*-bridged diiron phthalocyanine complexes (also known as mu-mitrido complexes) [105] are powerful oxidation catalysts [106], and therefore the **DiSO_2_Hex** substitution pattern both increases their solubility in reaction solvents and avoids the risk of catalyst degradation. In order to gain more insights into the catalytic mechanism of such oxidation reactions, the heteroleptic *N*-bridged diiron phthalocyanine complex **FePc-N-FePc(SO2Hex)8** (Figure 13B) was designed and prepared, demonstrating that the formation of the hyperoxo intermediate occurs on the electron-rich phthalocyanine macrocycle, which was confirmed by theoretical calculations [107].

Used in ambipolar gas sensors devices [108], **NiPc(SO2Hex)8** exhibited opposite electronic properties compared to **NiPc(SHex)8** (Figure 13C) when used in bilayer heterojunctions with LuPc_2_, which was useful for tailoring the properties of the device.

Finally, the A2B asymmetric derivative **ZnPc(SHex)6-NH_2_** was prepared to be covalently conjugated onto reduced graphene oxide (RGO) together with a porphyrin (Figure 13D) [109]. The resulting donor–π–acceptor graphene nanoconjugate undergoes photoinduced cascading electron and charge transfer from the porphyrin to RGO and then from RGO to the phthalocyanine, with long-lived charge separation. It also exhibited great nonlinear properties when nanosecond laser irradiated at 532 nm, with an NLO coefficient superior to 800 cm/GW.

## References

[1] De la Torre G, Martinez-Diaz MC, Torres T (1999). Synthesis of fused polynuclear systems based on phthalocyanine and triazolehemiporphyrazine units. Journal of Porphyrins and Phthalocyanines.

[2] Gürek AG, Bekaroğlu Ö (1994). Octakis(alkylthio)-substituted phthalocyanines and their interactions with silver(I) and palladium(II) ions. Journal of the Chemical Society, Dalton Transactions.

[3] Wöhrle D, Eskes M, Shigehara K, Yamada A (1993). A simple synthesis of 4,5-disubstituted 1,2-dicyanobenzenes and 2,3,9,10,16,17,23,24-octasubstituted phthalocyanine. Synthesis.

[4] Zorlu Y, İşci Ü, Ün İ, Kumru U, Dumoulin F (2013). Comparative structural analysis of 4,5- and 3,6-dialkylsulfanylphthalonitriles of different bulkiness. Structural Chemistry.

[5] Önal E, Okyay TM, Ekineker G, Işci Ü, Ahsen V (2018). Sulfanyl vs sulfonyl, 4,5- vs 3,6- position. How structural variations in phthalonitrile substitution affect their infra-red, crystallographic and Hirshfeld surface analyses. Journal of Molecular Structure.

[6] Sezer E, Ustamehmetoğlu B, Altuntaş Bayır Z, Çoban K, Kalkan A (2011). Corrosion inhibition effect of 4-(2-diethylamino-ethylsulfonyl)-phthalonitrile and 4,5-bis(hexylsulfonyl)-phthalonitrile. International Journal of Electrochemistry.

[7] Özkaya AR, Gürek AG, Gül A, Bekaroglu Ö (1997). Electrochemical and spectral properties of octakis(hexylthio)-substituted phthalocyanines. Polyhedron.

[8] Lux A, Rozenberg GG, Petritsch K, Moratti SC, Holmes AB (1999). A series of novel liquid crystalline octakis(alkylthio)-substituted phthalocyanines. Synthetic Metals.

[9] Basova TV, Gürek AG, Ahsen V (2002). Investigation of liquid-crystalline behavior of nickel octakisalkylthiophthalocyanines and orientation of their films. Materials Science and Engineering C.

[10] Basova T, Kol’tsov E, Gürek AG, Atilla D, Ahsen V (2008). Investigation of liquid-crystalline behaviour of copper octakisalkylthiophthalocyanine and its film properties. Materials Science and Engineering C.

[11] Basova T, Hassan A, Durmus M, Gürek AG, Ahsen V (2016). Liquid crystalline metal phthalocyanines: structural organization on the substrate surface. Coordination Chemistry Reviews.

[12] Paul S, Paul D, Basova T, Ray AK (2008). Studies of adsorption and viscoelastic properties of proteins onto liquid crystal phthalocyanine surface using quartz crystal microbalance with dissipation technique. Journal of Physical Chemistry C.

[13] Basova T, Paul S, Paul D, Vadgama P, Gürek AG (2008). Liquid crystalline phthalocyanine thin films as nanoscale substrates for protein adsorption. Journal of Bionanoscience.

[14] Paul S, Paul D, Basova T, Ray AK (2010). Characterisation of protein adsorption on different liquid crystal phthalocyaninethin films. IET Nanobiotechnology.

[15] Paolesse R, Nardis S, Monti D, Stefanelli M, Di Natale C (2017). Porphyrinoids for Chemical Sensor Applications. Chemical Reviews.

[16] Zhou R, Josse F, Göpel W, Öztürk ZZ, Bekaroğlu Ö (1996). Phthalocyanines as sensitive materials for chemical sensors. Applied Organometallic Chemistry.

[17] Gounden D, Nombona N, van Zyl WE (2020). Recent advances in phthalocyanines for chemical sensor, non-linear optics (NLO) and energy storage applications. Coordination Chemistry Reviews.

[18] Öztürk ZZ, Kılınç N, Atilla D, Gürek AG, Ahsen V (2009). Recent studies chemical sensors based on phthalocyanines. Journal of Porphyrins and Phthalocyanines.

[19] Klyamer D, Shutilov R, Basova T (2022). Recent advances in phthalocyanine and porphyrin-based materials as active layers for nitric oxide chemical sensors. Sensors.

[20] Kılınç N, Atilla D, Öztürk S, Gürek AG, Öztürk ZZ (2009). Oxidizing gas sensing properties of mesogenic copper octakisalkylthiophthalocyanine chemoresistive sensors. Thin Solid Films.

[21] Mashazi P, Antunes E, Nyokong T (2010). Probing electrochemical and electrocatalytic properties of cobalt(II) and manganese(III) octakis(hexylthio) phthalocyanine as self-assembled monolayers. Journal of Porphyrins and Phthalocyanines.

[22] Topal SZ, İşci Ü, Kumru U, Atilla D, Gürek AG (2014). Modulation of the electronic and spectroscopic properties of Zn(II) phthalocyanines by their substitution pattern. Dalton Transactions.

[23] Garifullin R, Erkal TS, Tekin S, Ortaç B, Gurek AG (2012). Encapsulation of a zinc phthalocyanine derivative in self-assembled peptide nanofibers. Journal of Materials Chemistry.

[24] Bharmoria P, Ghasemi S, Edhborg F, Losantos R, Wang Z (2022). Far-red triplet sensitized Z-to-E photoswitching of azobenzene in bioplastics. Chemical Science.

[25] Arslanoglu Y, Sevim AM, Hamuryudan E, Gul A (2006). Near-IR absorbing phthalocyanines. Dyes and Pigments.

[26] Canlıca M, Nyokong T (2011). Synthesis and photophysical properties of metal free, titanium, magnesium and zinc phthalocyanines substituted with a single carboxyl and hexylthio groups. Polyhedron.

[27] Mayukh M, Sema CM, Roberts JM, McGrath DV (2010). Solvent-free synthesis of soluble, near-IR absorbing titanyl phthalocyanine derivatives. Journal of Organic Chemistry.

[28] Ateş Turkmen T, Zeng L, Cui Y, Fidan I, Dumoulin F (2018). Effect of the substitution pattern (peripheral vs non-peripheral) on the spectroscopic, electrochemical, and magnetic properties of octahexylsulfanyl copper phthalocyanines. Inorganic Chemistry.

[29] Kılınç N, Sennik E, Atilla D, Gürek AG, Ahsen V (2013). Effect of ambient atmosphere on photoconductivity of TiO_2_ nanotube-CuPc heterojunction. Science of Advanced Materials.

[30] Dumoulin F, Zorlu Y, Ayhan MM, Hirel C, İsci Ü (2009). A first ABAC phthalocyanine. Journal of Porphyrins and Phthalocyanines.

[31] Chow SY, Ng DK (2016). Synthesis of an ABCD-type phthalocyanine by intramolecular cyclization reaction. Organic Letters.

[32] Nemykin VN, Dudkin SV, Dumoulin F, Hirel C, Gurek AG (2014). Synthetic approaches to asymmetric phthalocyanines and their analogues. Arkivoc.

[33] Kobayashi N, Kondo R, Nakajima S, Osa T (1990). New route to unsymmetrical phthalocyanine analogs by the use of structurally distorted subphthalocyanines. Journal of the American Chemical Society.

[34] Ekren SB, Dumoulin F, Musluoğlu E, Ahsen V, Güngör Ö (2019). A_3_B and ABAB aminophthalocyanines: building blocks for dimeric and polymeric constructs. Journal of Porphyrins and Phthalocyanines.

[35] Gürsoy S, Altuntaş Bayır Z, Hamuryudan E, Bekaroğlu Ö (2000). Synthesis and characterization of new unsymmetrically substituted phthalocyanines. Monatshefte für Chemie/Chemical Monthly.

[36] Canlıca M (2008). Synthesis and characterization of a new asymmetrical phthalocyanine with Zn(II), Ni(II) and Co(II). Asian Journal of Chemistry.

[37] Hamuryudan E (2006). Synthesis and solution properties of phthalocyanines substituted with four crown ethers. Dyes and Pigments.

[38] Arslanoğlu Y, Koca A, Hamuryudan E (2011). The synthesis and electrochemical study of novel phthalocyanines substituted with a crown ether and alkyl chains. Dyes and Pigments.

[39] Batat P, Bayar M, Pur B, Çoker E, Ahsen V (2016). The optical characterization of metal-mediated aggregation behaviour of amphiphilic Zn(II) phthalocyanines. Physical Chemistry Chemical Physics.

[40] Kalkan A, Koca A, Altuntaş Bayır Z (2004). Unsymmetrical phthalocyanines with alkynyl substituents. Polyhedron.

[41] Özçeşmeci İ, Kalkan Burat A, Altuntaş Bayır Z (2014). Synthesis and photophysical properties of novel unsymmetrical metal-free and metallophthalocyanines. Journal of Organometallic Chemistry.

[42] Altuntaş Bayır Z, Merey Ş, Hamuryudan E (2003). Metal-containing phthalocyanines substituted with one branched bulky moiety and six alkylthio groups. Monatshefte für Chemie/Chemical Monthly.

[43] Dincer HA, Gül A, Koçak MB (2004). A novel route to 4-chloro-5-alkyl-phthalonitrile and phthalocyanines derived from it. Journal of Porphyrins and Phthalocyanines.

[44] Uğur AL, Dincer HA, Erdoğmuş A (2012). Synthesis, photophysical and thermal studies of symmetrical and unsymmetrical zinc phthalocyanines. Polyhedron.

[45] Kulaç D, Bulut M, Altındal A, Özkaya AR, Salih B (2007). Synthesis and characterization of novel 4-nitro-2-(octyloxy)phenoxy substituted symmetrical and unsymmetrical Zn(II), Co(II) and Lu(III) phthalocyanines. Polyhedron.

[46] Burat AK, Karaoğlu HRP (2021). Photophysical properties of a newly synthesized unsymmetrically substituted zinc phthalocyanine. Journal of the Turkish Chemical Society Section A: Chemistry.

[47] Zhu YJ, Huang JD, Jiang XJ, Sun JC (2006). Novel silicon phthalocyanines axially modified by morpholine: synthesis, complexation with serum protein and in vitro photodynamic activity. Inorganic Chemistry Communications.

[48] Burat AK, Koca A, Lewtak JP, Gryko DT (2010). Synthesis, physicochemical properties and electrochemistry of morpholine-substituted phthalocyanines. Journal of Porphyrins and Phthalocyanines.

[49] Burat AK, Koca A, Lewtak JP, Gryko DT (2011). Preparation, electrochemistry and optical properties of unsymmetrical phthalocyanines bearing morpholine and tert-butylphenoxy substituents. Synthetic Metals.

[50] Dumrul H, Yüksel F (2013). Synthesis and characterization of novel symmetrical and asymmetrical substituted Zn(II) phthalocyanines. Polyhedron.

[51] Yüksel F, Atilla D, Ahsen V (2007). Synthesis and characterization of liquid crystalline unsymmetrically substituted phthalocyanines. Polyhedron.

[52] Bayda M, Dumoulin F, Hug GL, Koput J, Gorniak R (2017). Fluorescent H aggregates of an asymmetrically substituted mono-amino Zn(II) phthalocyanine. Dalton Transactions.

[53] Zucolotto Cocca LH, Ayhan MM, Gürek AG, Ahsen V, Bretonnière Y (2016). Mechanism of the Zn(II)phthalocyanines’ photochemical reactions depending on the number of substituents and geometry. Molecules.

[54] De la Torre G, Claessens CG, Torres T (2007). Phthalocyanines: old dyes, new materials. Putting color in nanotechnology. Chemical Communications.

[55] Schmidt AM, Calvete MJF (2021). Phthalocyanines: an old dog can still have new (photo)tricks. Molecules.

[56] Taquet JP, Frochot C, Manneville V, Barberi-Heyob M (2007). Phthalocyanines covalently bound to biomolecules for a targeted photodynamic therapy. Current Medicinal Chemistry.

[57] Kumru U, Ermeydan MA, Dumoulin F, Ahsen V (2008). Amphiphilic galactosylated phthalocyanines. Journal of Porphyrins and Phthalocyanines.

[58] Ermeydan MA, Dumoulin F, Basova TV, Bouchu D, Gürek AG (2010). Amphiphilic carbohydrate–phthalocyanine conjugates obtained by glycosylation or by azide–alkyne click reaction. New Journal of Chemistry.

[59] Dumoulin F, Lafont D, Boullanger P, Mackenzie G, Mehl GH (2002). Self-organizing properties of natural and related synthetic glycolipids. Journal of the American Chemical Society.

[60] Banoub J, Boullanger P, Lafont D (1992). Synthesis of oligosaccharides of 2-amino-2-deoxy sugars. Chemical Reviews.

[61] Rio S, Beau JM, Jacquinet JC (1991). Synthesis of glycopeptides from the carbohydrate-protein linkage region of proteoglycans. Carbohydrate Research.

[62] Lee BY, Park SR, Jean HB, Kim KS (2006). A new solvent system for efficient synthesis of 1,2,3-triazoles. Tetrahedron Letters.

[63] Nyokong T (2007). Effects of substituents on the photochemical and photophysical properties of main group metal phthalocyanines. Coordination Chemistry Reviews.

[64] Özgür N, Nar I, Gül A, Hamuryudan E (2015). A new unsymmetrical phthalocyanine with a single o-carborane substituent. Journal of Organometallic Chemistry.

[65] Bartelmess J, Ballesteros B, de la Torre G, Kiessling D, Campidelli S (2010). Phthalocyanine–pyrene conjugates: a powerful approach toward carbon nanotube solar cells. Journal of the American Chemical Society.

[66] Kabay N, Bozer BD, Öztürk Kiraz A, Baygu Y, Kara İ (2019). Synthesis, characterization and structural computational investigation of novel Zn(II) phthalocyanines containing peripheral anthracene moieties. Journal of Porphyrins and Phthalocyanines.

[67] Topkaya D, Dumoulin F, Ahsen V, İşci Ü (2014). Axial binding and host–guest interactions of a phthalocyanine resorcinarene cavitand hybrid. Dalton Transactions.

[68] Jimenez AJ, Krick Calderon RM, Rodriguez-Morgade MS, Guldi DM, Torres T (2013). Synthesis, characterization and photophysical properties of a melamine-mediated hydrogen-bound phthalocyanine–perylenediimide assembly. Chemical Science.

[69] Ragoussi ME, Ince M, Torres T (2013). Recent advances in phthalocyanine-based sensitizers for dye-sensitized solar cells. European Journal of Organic Chemistry.

[70] Urbani M, Ragoussi ME, Nazeeruddin MK, Torres T (2019). Phthalocyanines for dye-sensitized solar cells. Coordination Chemistry Reviews.

[71] Yıldız B, Güzel E, Akyüz D, Arslan BS, Koca A (2019). Unsymmetrically pyrazole-3-carboxylic acid substituted phthalocyanine-based photoanodes for use in water splitting photoelectrochemical and dye-sensitized solar cells. Solar Energy.

[72] Yıldız B, Ahmetali E, Arslan BS, Menges N, Nebioğlu M (2022). Effect of direct linkage of pyrazole carboxylic acid acceptor/anchoring group on the photovoltaic performance for phthalocyanine-sensitized solar cells. Dyes and Pigments.

[73] Yazıcı A, Ateş D, Bekaroğlu Ö, Kobayashi N (2006). Synthesis and characterization of novel azo-bridged Zn(II) and Co(II) bisphthalocyanines. Journal of Porphyrins and Phthalocyanines.

[74] Salan Ü, Kobayashi N, Bekaroğlu Ö (2009). Effect of peripheral substitution on the electronic absorption and magnetic circular dichroism (MCD) spectra of metal-free azo-coupled bisphthalocyanine. Tetrahedron Letters.

[75] Canlıca M, Altındal A, Özkaya AR, Salih B, Bekaroğlu Ö (2008). Synthesis, characterization, and electrochemical, and electrical measurements of novel 4,40-isopropylidendioxydiphenyl bridged bis and cofacial bis-metallophthalocyanines (Zn,Co). Polyhedron.

[76] Odabaş Z, Altındal A, Özkaya AR, Bulut M, Salih B (2007). Synthesis, characterization, and electrochemical, spectroelectrochemical and electrical measurements of novel ball-type four 1,10-methylenedinaphthalen-2-ol bridged metal-free, zinc(II) and cobalt(II), and metal-free clamshell phthalocyanines. Polyhedron.

[77] Ceyhan T, Altındal A, Özkaya AR, Çelikbıçak Ö, Salih B (2007). Synthesis, characterization and electrochemical properties of novel metal free and zinc(II) phthalocyanines of ball and clamshell types. Polyhedron.

[78] Ozan N, Bekaroğlu Ö (2003). Synthesis and characterization of a triazine containing three phthalocyanines. Polyhedron.

[79] Ceyhan T, Korkmaz M, Kutluay T, Bekaroğlu Ö (2004). Synthesis, characterization and EPR spectroscopy of novel s-triazines bearing three oxygen-linked phthalocyanines. Journal of Porphyrins and Phthalocyanines.

[80] Şen P, Duludağ F, Salih B, Özkaya AR, Bekaroğlu Ö (2011). Synthesis and electrochemical, electrochromic and electrical properties of novel s-triazine bridged trinuclear Zn(II), Cu(II) and Lu(III) and a tris double-decker Lu(III) phthalocyanines. Synthetic Metals.

[81] Özer M, Altındal A, Özkaya AR, Bulut M, Bekaroğlu Ö (2005). Synthesis, characterization, and electrical, electrochemical and gas sensing properties of a novel cyclic borazine derivative containing three phthalocyaninato zinc(II) macrocycles. Synthetic Metals.

[82] Ceyhan C, Altındal A, Erbil MK, Özer Bekaroğlu Ö (2006). Synthesis, characterization, conduction and gas sensing properties of novel multinuclear metallo phthalocyanines (Zn, Co) with alkylthio substituents. Polyhedron.

[83] Kolb HC, Finn MG, Sharpless KB (2001). Click chemistry: diverse chemical function from a few good reactions. Angewandte Chemie International Edition.

[84] Dumoulin F, Ahsen V (2011). Click chemistry: the emerging role of the azide-alkyne Huisgen dipolar addition in the preparation of substituted tetrapyrrolic derivatives. Journal of Porphyrins and Phthalocyanines.

[85] Acherar S, Colombeau L, Frochot C, Vanderesse R (2015). Synthesis of porphyrin, chlorin and phthalocyanine derivatives by azide-alkyne click chemistry. Current Medicinal Chemistry.

[86] Gonidec M, Biagi R, Corradini V, Moro F, De Renzi V (2011). Surface supramolecular organization of a terbium(III) double-decker complex on graphite and its single molecule magnet behavior. Journal of the American Chemical Society.

[87] Ishikawa N, Sugita M, Ishikawa T, Koshihara SY, Kaizu Y (2003). Lanthanide double-decker complexes functioning as magnets at the single-molecular level. Journal of the American Chemical Society.

[88] Gürek AG, Ahsen V, Luneau D, Pécaut P (2001). Synthesis, structure, spectroscopic properties, and magnetic properties of an octakis(alkylthio)-substituted lutetium(III) bisphthalocyanine. Inorganic Chemistry.

[89] De Cian A, Moussavi M, Fischer J, Weiss R (1985). Synthesis, structure, and spectroscopic and magnetic properties of lutetium (III) phthalocyanine derivatives: LuPc_2_.CH_2_Cl_2_ and [LuPc(OAc)(H_2_O)_2_].H_2_O.2CH_3_OH. Inorganic Chemistry.

[90] Gürek AG, Basova T, Luneau D, Lebrun C, Kol’tsov E (2006). Synthesis, structure, and spectroscopic and magnetic properties of mesomorphic octakis(hexylthio)-substituted phthalocyanine rare-earth metal sandwich complexes. Inorganic Chemistry.

[91] Basova T, Jushina I, Gürek AG, Ahsen V, Ray AK (2008). Use of the electrochromic behaviour of lanthanide phthalocyanine films for nicotinamide adenine dinucleotide detection. Journal of the Royal Society Interface.

[92] Rana S, Jiang J, Korpany KV, Mazur U, Hipps KW (2021). STM investigation of the Y[C_6_S-Pc]_2_ and Y[C_4_O-Pc]_2_ complex at the solution–solid interface: substrate effects, submolecular resolution, and vacancies. The Journal of Physical Chemistry C.

[93] Ayhan MM, Singh A, Hirel C, Gürek AG, Ahsen V (2012). ABAB homoleptic bis (phthalocyaninato)lutetium(III) complex: toward the real octupolar cube and giant quadratic hyperpolarizability. Journal of the American Chemical Society.

[94] Ayhan MM, Singh A, Jeanneau E, Ahsen V, Zyss J (2014). ABAB homoleptic bis(phthalocyaninato)lanthanide(III) complexes: original octupolar design leading to giant quadratic hyperpolarizability. Inorganic Chemistry.

[95] Dabak S, Bekaroglu Ö (1997). Synthesis of phthalocyanines crosswise-substituted with two alkylsulfanyl and two amino groups. New Journal of Chemistry.

[96] Ekren SB, Dumoulin F, Musluoğlu E, Ahsen V, Güngör Ö (2019). A_3_B and ABAB aminophthalocyanines: building blocks for dimeric and polymeric constructs. Journal of Porphyrins and Phthalocyanines.

[97] Ayhan MM, Zorlu Y, Gökdemir Ö, Gürek AG, Dumoulin F (2014). Optimized synthesis and crystal growth by sublimation of 1,3,3-trichloroisoindolenines, key building blocks for crosswise phthalocyanines. CrystEngComm.

[98] Alpugan S, Isci U, Albrieux F, Hirel C, Gurek AG (2014). Expeditious selective access to functionalized platforms of A7B-type heteroleptic lanthanide double-decker complexes of phthalocyanine. Chemical Communications.

[99] Huang C, Wang K, Sun J, Jiang J (2014). Planar binuclear phthalocyanine-containing sandwich-type rare-earth complexes: synthesis, spectroscopy, electrochemistry, and NLO properties. European Journal of Inorganic Chemistry.

[100] Lu G, Kong X, Sun J, Zhang L, Chen Y (2017). Solution-processed single crystal microsheets of a novel dimeric phthalocyanine-involved triple-decker for high-performance ambipolar organic field effect transistors. Chemical Communications.

[101] Abdurrahmanoğlu Ş, Özkaya AR, Bulut M, Bekaroğlu Ö (2004). Synthesis, characterization, and electrochemical and electrochromic properties of sandwich dilutetium tetraphthalocyanine. Dalton Transactions.

[102] Şen P, Dumludağ F, Salih B, Özkaya AR, Bekaroğlu Ö (2011). Synthesis and electrochemical, electrochromic and electrical properties of novel s-triazine bridged trinuclear Zn(II), Cu(II) and Lu(III) and a tris double-decker Lu(III) phthalocyanines. Synthetic Metals.

[103] Sastre A, del Rey B, Torres T (1996). Synthesis of novel unsymmetrically substituted push-pull phthalocyanines. The Journal of Organic Chemistry.

[104] Zhang Y, Ma P, Zhu P, Zhang X, Gao Y (2011). 2,3,9,10,16,17,23,24-Octakis(hexylsulfonyl)phthalocyanines with good n-type semiconducting properties. Synthesis, spectroscopic, and electrochemical characteristics. Journal of Materials Chemistry.

[105] İşci Ü, Dumoulin F, Sorokin AB, Ahsen V (2014). N-bridged dimers of tetrapyrroles complexed by transition metals: syntheses, characterization methods, and uses as oxidation catalysts. Turkish Journal of Chemistry.

[106] İşci Ü, Afanasiev P, Millet JMM, Kudrik EV, Ahsen V (2009). Preparation and characterization of μ-nitrido diiron phthalocyanines with electron-withdrawing substituents: application for catalytic aromatic oxidation. Dalton Transactions.

[107] İşci Ü, Faponle AS, Afanasiev P, Albrieux F, Briois V (2015). Site-selective formation of an iron(iv)–oxo species at the more electron-rich iron atom of heteroleptic μ-nitrido diiron phthalocyanines. Chemical Science.

[108] Şahin Z, Meunier-Prest R, Dumoulin F, Kumar A, İsci Ü (2021). Tuning of organic heterojunction conductivity by the substituents’ electronic effects in phthalocyanines for ambipolar gas sensors. Sensors and Actuators B: Chemical.

[109] Fu L, Li H, Fang Y, Guan Z, Wei Z (2023). Cascading electron transfer and photophysics in a donor-π-acceptor graphene nanoconjugate. Nano Research.

